# How healthy are food and beverage products promoted by TikTok influencers?

**DOI:** 10.1017/S136898002510181X

**Published:** 2026-01-07

**Authors:** Roxanne Dupuis, Aviva A. Musicus, Omni Cassidy, Marie A. Bragg

**Affiliations:** 1 Department of Population Health, NYU Grossman School of Medicine, New York, NY, USA; 2 NYU Food Environment and Policy Research Coalition, New York, NY, USA; 3 Center for Science in the Public Interest, Washington, DC, USA; 4 Department of Nutrition, Harvard TH Chan School of Public Health, Boston, MA, USA; 5 Marketing Department, NYU Stern School of Business, New York, NY, USA

**Keywords:** Food marketing, Nutrient Profile Index, Social media

## Abstract

**Objective:**

To evaluate the healthfulness of the food/beverage products featured by TikTok influencers whose audiences include millions of adolescents.

**Design:**

In a cross-sectional study, we collected the maximum available up to 100 videos from the top 100 TikTok influencers in the USA – based on views, likes, comments and shares – in July 2022. For each video, we identified the most prominent food/beverage product featured. We used the Nutrient Profile Index (NPI) to classify food products as healthy/unhealthy. We grouped beverages by category.

**Setting:**

TikTok

**Participants:**

N/A

**Results:**

Our sample included 8871 videos, 1360 (15·3 %) of which featured at least one food (*n* 755, 55·5 %), beverage (*n* 580, 42·6 %) or dietary supplement (*n* 25, 1·8 %). Mean NPI score for foods was 54·73 (sd 19·95). Most foods (58 %) were considered unhealthy, with a 20-percentage-point difference between branded (70·8 %) and unbranded (50·8 %) foods. Alcohol (*n* 154, 26·6 %) and energy drinks (*n* 149, 25·7 %) were the most featured beverages overall. Among branded beverages, energy drinks were the largest category (*n* 148, 38·9 %). Among unbranded beverages, alcoholic drinks were the largest category (*n* 73, 36·5 %).

**Conclusions:**

More than half of the foods promoted by TikTok influencers were considered unhealthy, and most beverages featured were alcoholic and energy drinks. Many foods and a large share of alcoholic beverages were unbranded, either reflecting genuine influencer preferences or potentially masking the true extent of commercial marketing. Given the reach of influencers, including millions of adolescents, stronger regulations are needed for social media platforms, influencers and brands to protect consumers from undue harm from food/beverage marketing.

Food and beverage marketing is a powerful force shaping eating norms, attitudes, behaviours and health^(1–3)^. Food/beverage brands are increasingly marketing their products on social media (SM)^(4,5)^. This is particularly problematic for American adolescents who spend almost 5 h each day on SM, where they may be exposed to high volumes of unhealthy food ads^(6)^.

TikTok – an SM application that features short videos that play automatically and rely on a complex (and obscure) algorithm to promote content – is currently one of the most popular platforms among adolescents^(7)^. Sixty-three per cent of adolescents in the USA report having used TikTok at least once, and 57 % report using it every day^(7)^. Even though TikTok is intended for adolescents aged ≥ 13 years – and some states have implemented stricter age eligibility and verification policies^(8,9)^ – younger adolescents have found ways to circumvent age restrictions. In 2020, one-third of TikTok users were aged ≤ 14 years^(10)^.

Adolescents are continuously exposed to food/beverage ads on SM^(11)^. In one study, 278 Canadian teenagers captured 1392 teen-targeted food ads from Instagram, Snapchat, TikTok and YouTube over a 7-d period^(12)^. In another study, Australian adolescents were exposed to 17·4 food/beverage ads per hour they spent online^(13)^. Research also indicates that adolescents are more likely to engage with food ads that have many *v*. few likes^(14)^– a key TikTok feature. Like traditional marketing, digital and SM marketing of unhealthy food/beverage products has negative impacts on health^(15,16)^.

Food marketing on TikTok – combined with its popularity among adolescents – raises several concerns. For example, current industry-led initiatives to restrict marketing to youth, like the Children’s Food and Beverage Advertising Initiative (CFBAI), only extend to adolescents under the age of 13 years^(17)^. Additionally, companies participating in CFBAI primarily market products of poor nutritional quality, as measured by nutrition criteria used by government agencies in the USA and UK^(18,19)^, meaning millions of young people are exposed to unhealthy food marketing on SM.

One popular marketing strategy on SM is to promote products through influencers. Unlike traditional celebrities (e.g. actors), influencers are seen as relatable, and their product endorsements are often taken as word-of-mouth recommendations^(20)^. A study assessing exposure to food/beverage promotions on SM among Australian adolescents found that 26 % of promotions were on celebrity or influencer accounts^(21)^. Additional studies have found that SM influencers, including celebrities, overwhelmingly promote unhealthy products^(22–24)^. Evidence of the effect of influencer marketing on consumption is emerging, with positive to null results^(16,25)^. For example, randomised trials among children aged 9–11 years indicate that influencer marketing of unhealthy foods leads to increased snack intake^(26,27)^.

In a recent study, we found that 15 % of the videos from the top 100 TikTok influencers in the USA in July 2022 featured at least one food/beverage product, and that nearly half of those products (48 %) featured a food/beverage brand^(24)^. Most videos featuring a brand (69 %) did not contain any disclosures of brand sponsorships. Among videos that contained disclosures, influencers used ten different types of disclosures, many of which were ambiguous about paid sponsorships (e.g. #[brandname] in the caption)^(28)^. The lack of disclosures and the use of ambiguous disclosures leave viewers guessing as to whether they watched a paid product placement or an influencer’s personal preference. While the US Federal Trade Commission regulates influencer marketing^(29)^, gaps in the regulatory framework allow influencers to circumvent any ramifications when posting by not acknowledging sponsored content. Building on this study, we sought to evaluate the healthfulness of the food/beverage products featured on the accounts of top influencers, framed within the context of known youth engagement with TikTok.

## Methods

### Data collection and classification

The research team screen-recorded up to 100 TikTok videos from the top 100 influencers in the USA overall (i.e., across all users, a significant portion of which are adolescents) – identified using HypeAuditor, an influencer marketing platform – as of 1 July 2022. HypeAuditor uses data on views, likes, comments and shares to create the list.

**Figure 1.**
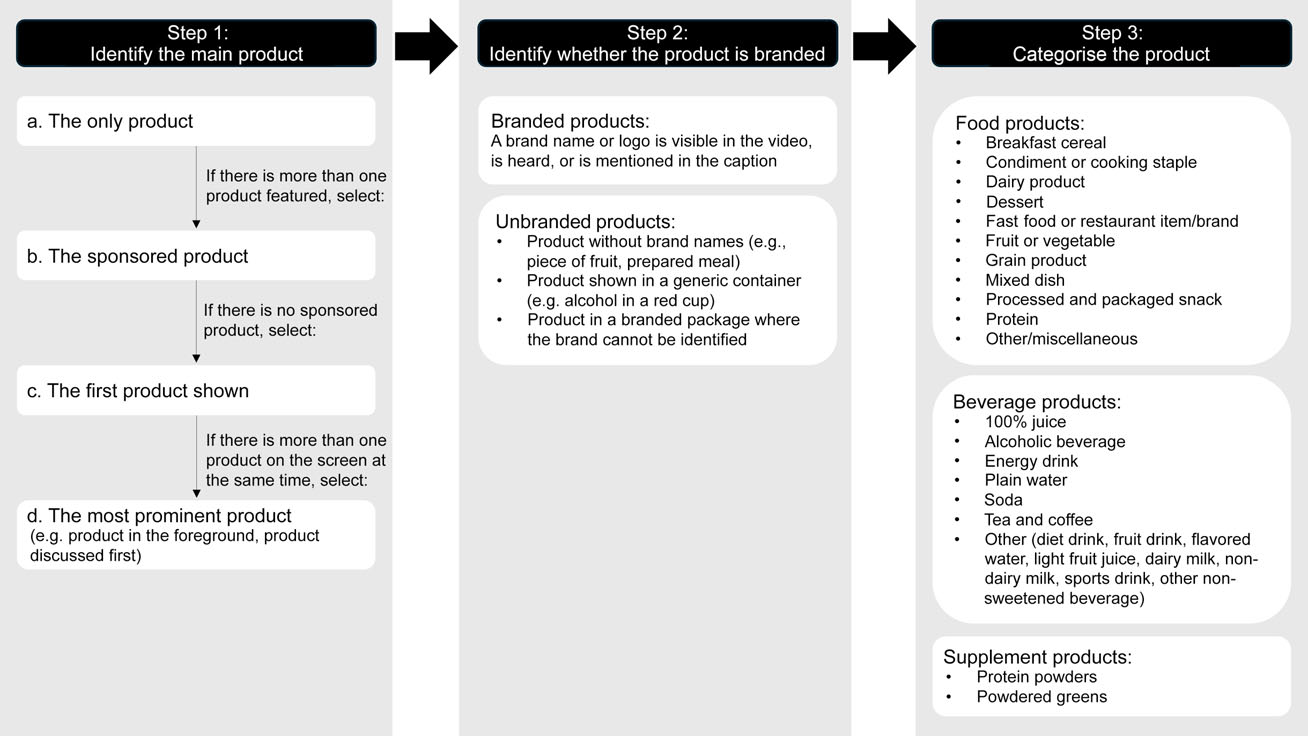
Steps to identify products and categorise them. To categorise food products, we identified products based on food groups and added categories for processed or packaged items (e.g. breakfast cereals, packaged snacks, and condiments and cooking staples) and for prepared foods (e.g. mixed dishes and desserts)^(47)^. We categorised foods from restaurants into their own category. For beverage products, we used the Rudd Center Children’s Drinks categories and added alcoholic beverages and plain water^(48)^. We combined sweetened and unsweetened coffee and tea into one category and collapsed some categories for which there were only a small number of items. These categories have been used in other food and beverage marketing evaluations, including on digital platforms.^(33,34,36)^

For each TikTok, we identified if a food/restaurant, beverage or supplement was featured and, if so, categorised the main product featured (Figure 1)^(28)^.

### Product scoring

To ascertain the healthfulness of food products, we used the Nutrient Profile Index (NPI). NPI scores are derived from the Nutrient Profile Model (NPM), a validated assessment developed to identify and regulate marketing of unhealthy food to children in the UK^(30–32)^. NPM considers the amount of specific macro- and micro-nutrients and the proportion of fruits, vegetables and/or nuts per serving size. NPM scores were converted into NPI scores for ease of interpretation – a protocol used in prior studies^(33–35)^ – with values ranging from 1 (least healthy) to 100 (most healthy), using the following formula: −2 × NPM score + 70^(36)^. An NPI score of ≥ 64, equivalent to an NPM score of ≤ 3 – the cut-off for advertising to children in the UK – is considered healthy^(36)^.

For branded/packaged foods, we extracted nutrition information from the official product websites. If flavour, for example, was not specified, we selected popular or frequently bought varieties (e.g. products that appeared on the home page or featured as customer ‘favourites’). We assumed that foods contained at least 50 % fruits, vegetables or nuts if these were listed in the first five ingredients. Because NPM calculates a score based on a serving size in grams, we used MenuStat 2022 data (https://catalog.data.gov/dataset/dohmh-menustat) or the USDA Food Data Central database (https://fdc.nal.usda.gov/index.html) to estimate weight for items for which serving size weight was not readily available.

For unspecified foods from a restaurant (e.g. McDonald’s combo meal), we extracted NPI scores from the Rudd Center’s Fast Food FACTS Report^(37)^. If information was not available in the report, we assessed nutrition information for the top five food products from the brand/company’s website and averaged their scores. For fast-food restaurant products, we used the top five combo meals using defaults as advertised on the website or by following the protocol used by Vercammen et al.^(38)^.

For fruits and vegetables, mixed dishes (e.g. lasagna) and unbranded foods (e.g. generic oat ‘O’ cereal), we used the USDA FoodData Central database. If we could not find a typical recipe for an item or find any nutrition information, we omitted the food from the NPI analysis. We noted any assumptions made when calculating scores.

Because NPM and NPI are not as reliable in evaluating beverages, we opted to report product categories.

### Statistical analysis

We report descriptive statistics. All analyses were completed in R version 4.4.1.

## Results

Our final sample included 8871 videos, 1360 (15·3 %) of which featured at least one food product, beverage or dietary supplement. Close to half (47·6 %) of the products were branded (Table 1).

**Table 1.** Product categories and classification

	All products (*n* 1360)		Branded products (*n* 648)		Unbranded products (*n* 712)	
	*n*	%	*n*	%	*n*	%
Product category						
Food	755	55·5	245	37·8	510	71·6
Beverage	580	42·6	380	58·6	200	28·1
Dietary supplement	25	1·8	23	3·5	2	0·3
Food categories						
Breakfast cereal	16	2·1	9	3·7	7	1·3
Condiment or cooking staple	29	3·8	17	7·0	12	2·3
Dairy product	21	2·7	6	2·5	15	2·9
Dessert	113	14·7	6	2·5	107	20·3
Fast-food/restaurant item/brand	116	15·1	81	33·2	35	6·7
Fruit/vegetable	99	12·9	4	1·6	95	18·1
Grains	38	4·9	9	3·7	29	5·5
Mixed dish	71	9·2	8	3·3	63	12·0
Processed/packaged snack	162	21·0	95	38·9	67	12·7
Protein	79	10·3	6	2·5	73	13·9
Other	26	3·4	3	1·2	23	4·4
NPI classification for food products^*^						
Healthy	281	42·0	70	29·2	211	49·2
Unhealthy	388	58·0	170	70·8	218	50·8
Beverage categories						
100 % juice	9	1·6	2	0·5	7	3·5
Alcoholic beverage	154	26·6	81	21·3	73	36·5
Energy drink	149	25·7	148	38·9	1	0·5
Plain water	67	11·6	32	8·4	35	17·5
Soda	43	7·4	35	9·2	8	4·0
Tea and coffee	78	13·4	53	13·9	25	12·5
Other	80	13·8	29	7·6	51	25·5

NPI, Nutrient Profile Index.

* Only 669 food products and restaurants are included in this analysis. We could not find the necessary nutrition information (e.g. because of unknown recipe) for eighty-six products.

Overall, there was a large variety of food products featured. The largest category was processed or packaged snacks (*n* 162, 21·0 %), followed by fast-food/restaurant items/brands (*n* 116, 15·41%) and desserts (*n* 113, 14·17%). Among posts featuring branded foods, the top three product categories were processed or packaged snacks (*n* 95, 38·9 %), fast-food/restaurant items/brands (*n* 81, 33·2 %) and condiments or cooking staples (*n* 17, 7·0 %). Among posts featuring unbranded foods, the top three product categories were desserts (*n* 107, 20·3 %), fruits and vegetables (*n* 95, 18·1 %) and protein (*n* 73, 13·9 %).

We calculated NPI scores for 669 (88·6 %) of the food products. Mean NPI score was 54·73 (sd 19·95), with an eight-point difference between branded (mean 49·58, sd 17·80) and unbranded (mean 57·61, sd 20·52) food products. Most foods (58 %) were considered unhealthy, with a 20-percentage-point difference between branded (70·8 %) and unbranded (50·8 %) foods.

Across all posts featuring beverage products, the most featured were alcoholic beverages (*n* 154, 26·6 %) and energy drinks (*n* 149, 25·7 %). Similarly, alcoholic beverages and energy drinks made up more than half of the branded beverages (*n* 81, 21·3 % and *n* 148, 38·9 %, respectively). Alcoholic beverages were also the largest category among unbranded products (*n* 73, 36·5 %), followed by ‘other’ beverages (*n* 51, 25·5 %).

## Discussion

Among the 1360 TikTok videos that featured food/beverage products from the top 100 influencers in the USA in July 2022, the largest beverage category was alcoholic drinks (26·6 %), almost half of which were classified as ‘unbranded.’ A high share of unbranded alcohol could underestimate the true extent of commercial alcohol promotion, since brand visibility may not always be captured. Relatedly, most food products were considered unhealthy (58·0 %), and a higher proportion of branded foods were considered unhealthy (70·8 %) compared to unbranded foods (50·8 %). Importantly, however, a large share of the unbranded food products could not be analysed for nutritional quality. It is possible that such food content represents the organic interests of influencers and is truly not a form of food marketing.

These findings add to the small, but growing, literature on the healthfulness of food/beverage products promoted by influencers. A study of the most popular influencers among Canadian adolescents by Amson et al. found that, across three platforms (including TikTok), restaurants were the most frequently featured food/beverage products (24 % of posts featuring food/beverage products), and most products were unhealthy^(24)^. In our study, fast-food/restaurant items/brands made up 15·1 % of the posts featuring food items, the second largest food category after processed/packaged snacks. The differences between our findings may reflect our different audiences – Amson et al. reviewed the posts by the top three influencers among Canadian adolescents across multiple platforms, while we reviewed the posts of the top 100 influencers overall on TikTok in the USA. Finally, a study of the content of food/beverage-related Instagram posts by highly followed traditional celebrities found that the most featured foods were snacks and sweets (37 %), while the most featured beverages contained alcohol (51 %)^(22)^. Overall, 61 % of the foods featured were considered unhealthy^(22)^. Our findings regarding the top TikTok influencers – many of whom would also be considered traditional celebrities – were similar, with the largest food category being processed/packaged snacks (21·0 % of foods) and the largest beverage category being alcoholic drinks (26·6 % of beverages).

While we did not have any data on the demographic profiles of the audiences for the top 100 influencers, we assumed that their overall popularity was indicative of their reach across TikTok users, a significant portion of whom are adolescents. In this respect, these trends in featuring or promoting primarily unhealthy food products, alcoholic beverages and energy drinks is particularly concerning. None of these products should be marketed to adolescents^(5,32,39,40)^, who may be uniquely susceptible to influencer and SM marketing^(4)^. Research indicates that, for adolescents, the aesthetic of an SM post, even on a traditional advertisement, is associated with a higher rating for that post^(41)^. Ads from influencers, specifically, blur the line between marketing and entertainment^(20)^, in addition to playing into adolescents’ desire to fit in with peers. Neuroimaging studies, for example, have shown that not only do adolescents like SM content with many *v*. few likes, but also that viewing content with many likes increases activation of the reward processing regions of the brain^(42)^. In combination, this research supports stronger regulation of food marketing and SM use among adolescents. Some states have proposed and enacted a range of policies to address these issues, including age restrictions, warning labels and the prevention of targeted marketing to minors on SM^(43–45)^. It will be important to evaluate whether such policies reduce exposure to unhealthy food/beverage products on SM.

This study has some limitations. We only included one product per video. In reality, each video could feature multiple products with different nutritional profiles. For many of the foods, we had to make assumptions about the recipe (e.g. lasagna), the specific flavour (e.g. tub of ice cream) or the specific items (e.g. fast-food takeout bag) featured to calculate the NPI. This also resulted in missing NPI data for 11·4 % (*n* 86) of foods, most of which were unbranded (*n* 81). Our sample included few dietary supplements. The ones identified were primarily powders (e.g. whey protein and greens) to add to foods and drinks. It is possible that other types of supplements (e.g. capsules) were not captured. It is also possible that the market for dietary supplements was not as saturated in July 2022 as it is now^(46)^.

Given the reach of influencers and the fact that adolescents spend considerable time on SM, stronger regulations are needed for platforms, influencers and brands to protect adolescents from undue harm from food/beverage marketing.
